# A Regional Model for Malaria Vector Developmental Habitats Evaluated Using Explicit, Pond-Resolving Surface Hydrology Simulations

**DOI:** 10.1371/journal.pone.0150626

**Published:** 2016-03-22

**Authors:** Ernest Ohene Asare, Adrian Mark Tompkins, Arne Bomblies

**Affiliations:** 1 Department of Physics, Kwame Nkrumah University of Science and Technology, Kumasi, Ghana; 2 Abdus Salam International Centre for Theoretical Physics (ICTP), Trieste, Italy; 3 School of Engineering, University of Vermont, 33 Colchester Ave, Burlington, VT 05405, United States of America; University of Catania, ITALY

## Abstract

Dynamical malaria models can relate precipitation to the availability of vector breeding sites using simple models of surface hydrology. Here, a revised scheme is developed for the VECTRI malaria model, which is evaluated alongside the default scheme using a two year simulation by HYDREMATS, a 10 metre resolution, village-scale model that explicitly simulates individual ponds. Despite the simplicity of the two VECTRI surface hydrology parametrization schemes, they can reproduce the sub-seasonal evolution of fractional water coverage. Calibration of the model parameters is required to simulate the mean pond fraction correctly. The default VECTRI model tended to overestimate water fraction in periods subject to light rainfall events and underestimate it during periods of intense rainfall. This systematic error was improved in the revised scheme by including the a parametrization for surface run-off, such that light rainfall below the initial abstraction threshold does not contribute to ponds. After calibration of the pond model, the VECTRI model was able to simulate vector densities that compared well to the detailed agent based model contained in HYDREMATS without further parameter adjustment. Substituting local rain-gauge data with satellite-retrieved precipitation gave a reasonable approximation, raising the prospects for regional malaria simulations even in data sparse regions. However, further improvements could be made if a method can be derived to calibrate the key hydrology parameters of the pond model in each grid cell location, possibly also incorporating slope and soil texture.

## Introduction

The availability of water for larvae development is a key determinant of mosquito density [1, 2]. Mosquitoes exploit diverse habitats for their oviposition though species have habitat-type preferences [1, 3–8]. For instance, the two key African malaria vectors, *Anopheles gambiae sensu stricto* and *Anopheles arabiensis* prefer small temporary sun-lit pools for oviposition [9–11] although they also thrive in other water bodies [3, 5, 8]. These temporary sun-lit pools typically have higher water temperatures, which shortens the length of the aquatic stage development of mosquitoes. Due to their size they are also prone to desiccation before larvae emerge as adults, implying that the temporal dynamics of small ponds is also important. For instance, Himeidan et al. [12] found hilltop habitats to be unproductive, containing few *Anopheline* larvae but with zero pupation rate as result of habitat instability. The availability, area coverage and persistence of temporary surface water (which serves as developmental habitat for gravid mosquitoes) are tied with depth, intensity and frequency of rainfall as well as local hydrological conditions.

Attempts to link rainfall incidence to malaria vector abundance and disease incidence have yielded varied results in different geographical locations. For instance, the 1997 El Ni*ň*o southern oscillation (ENSO) caused an increase in rainfall in parts of eastern Africa leading to a malaria epidemic in southwest Uganda [13], but conversely a reduction in malaria cases was observed in the Usambara Mountains of Tanzania [14], indicating that the relationship between rainfall and malaria transmission is nonlinear and complex. In Botswana, for example, Thomson et al. [15] developed a malaria early warning system based on multi-model ensemble prediction of precipitation and found that the relationship between November-February precipitation and the anomaly in malaria incidence is best explained by a quadratic relationship with malaria incidence decreasing once rainfall exceeded a certain threshold. In Malawi, Lowe et al. [16] found a similar quadratic relation. Kelly-Hope et al. [17] observed a weak correlation between precipitation and abundance of mosquito vectors with a correlation coefficient (r^2^) of 0.246 and 0.315 for *An. gambiae s.s* and *An. arabiensis* respectively. Similarly, Molineaux and Gramiccia [18] found a poor correlation between mosquito abundance and seasonal rainfall using data from Garki district in northern Nigeria. In addition, in Banizoumbou village in southwestern Niger, Bomblies [19] showed that temporal patterns of individual rainfall events are related to mosquito abundance, partially explaining previously observed poor correlations which typically consider monthly or seasonal total precipitation. The nonlinear relationship of mosquito abundance to precipitation is poorly understood and may be partially due to intense rainfall reducing larvae density by flushing first stage larvae [20]. Patz [21] observed an improvement in predicting *An. gambiae* biting rate from 8% with raw precipitation to 45% with modelled soil moisture, used as a proxy for pond availability. In a related study in South Africa, Montosi et al. [22] found that soil moisture predicts better sub-seasonal variability in malaria cases relative to rainfall and temperature. Shaman et al. [23] found a positive association between modelled local surface wetness with ≈ 10 days time lag and abundance of *Anopheles walkeri* and *Aedes vexans* in the eastern United States.

It is clear that for dynamical malaria models to accurately reproduce variations in malaria associated with rainfall, then they need to be able to include a representation of the surface hydrology correctly. Thus rainfall would drive a simple model for breeding site availability, which ultimately determines vector density. The rainfall may be obtained from a nearby station if available, but if malaria is to be modelled regionally recourse to satellite-merged retrieval products may be necessary due to sparse station data availability.

Dynamical mathematical models for mosquito density or malaria transmission have incorporated representations of the surface hydrology that vary in complexity. Depinay et al. [24] introduced a local scale dynamical model designed to explicitly model individual breeding sites to simulate mosquito population dynamics. Hoshen and Morse [25] relate the oviposition rate to the 10 day rainfall rate in the Liverpool Malaria Model (LMM). Lunde et al. [26] parametrized surface hydrology as a function of river length and soil moisture based on the assumption that potential habitats are located within the vicinity of rivers and lakes. Their Open Malaria Warning (OMaWa), designed to be run on a large scale, may have limited application in areas of relatively flat topography where habitats are only rain-fed and can be located far away from permanent water bodies. Another recently introduced regional scale dynamical malaria model, the vector-borne disease community model of the International Centre for Theoretical Physics, Trieste (VECTRI; Tompkins and Ermert [27]) uses a simple surface hydrology parametrization that models the evolution of the fractional water coverage within each grid cell.

Observations are required to evaluate and improve treatment of surface hydrology, however the small spatial scale of breeding habitats confounds such efforts. Field data can provide useful measurements of specific breeding sites, but are unable to assess the statistics of all ponds over a large area. At the same time, presently no evaluated and validated remotely sensed retrieval products exist for the coverage of small scale ponds.

A possible method to progress is to employ ultra-high resolution, explicit hydrology simulations as an intermediate step. Explicit simulations, which require mapping out the topography, land cover and soil texture at O(m) scale can only be conducted over areas of O(km) scale, due to the data input requirements which require manual mapping of an area, and additionally due to the computational cost. However, such explicit simulations for an O(km)-scale “patch” can be validated by isolated in situ measurements at this village scale, and then subsequently be used to provide O(km)-scale pond coverage evolution statistics to evaluate a simple scheme of a model such as VECTRI, which can potentially be applied on regional scales.

One explicit hydrological model, HYDREMATS, has been applied previously to simulate all individual ponds for a domain of several kilometres, which was validated at specific sites within the domain using measurements taken from a selection of ponds [28]. For local, village scale modelling, Bomblies et al. [28] introduced the high-resolution Hydrology, Entomology, and Malaria Transmission Simulator (HYDREMATS). HYDREMATS runs with 10 meter spatial scale grid-cells to explicitly simulate pool formation and persistence time that control aquatic stage development of mosquito for each individual pond. In order to set up this model, Banizoumbou village in southwest Niger was manually mapped at this 10 m scale using survey-grade differential GPS instrumentation. The model simulated daily water depth and showed good agreement with observations, predicting seasonal and sub-seasonal mosquito abundance [28]. Bomblies et al. [29] using HYDREMATS found good agreement with observed interannual variability in mosquito abundance between two villages located 30 km apart, but with contrasting local hydrological and environmental conditions. Furthermore, HYDREMATS has been used to assess the impact of environmental management in malaria control [30] and the sensitivity of the model to various climate change scenarios has been evaluated [31].

The objectives of this study are to use the statistics of all ponds aggregated over the entire HYDREMATS domain to evaluate the pond parametrization used in VECTRI [27]. A modified version of the scheme that attempts to address some shortcomings of the default VECTRI model is also presented and evaluated. Finally, we examine how the simulation of mosquito density by the simple bulk model of VECTRI compares to the detailed agent-based treatment of the HYDREMATS model.

Rain gauge networks in most malaria endemic regions are sparse and therefore available satellites estimates of rainfall can be useful to drive these surface hydrology schemes. Recently, Yamana and Eltahir [32], found similarities in mosquito population and malaria transmission simulated by HYDREMATS when driven by surface observations or the Climate Prediction Center morphing technique (CMORPH) rainfall estimates for Banizoumbou village in Niger. Their results revealed that in the absence of ground observations, satellite rainfall estimates may be used to drive malaria models. The second objective of this study is therefore to assess if replacing ground-based in situ rainfall measurements by remotely sensed rainfall data is significantly detrimental to the hydrology simulations. The results will indicate whether satellite products could be used to drive VECTRI so as to get real time prediction of malaria transmission on a regional scale.

## Method and Data

### Data for study Region

The study region, Banizoumbou village in southwestern Niger (13° 31′, 2° 39′), was mapped out at 10 m resolution and in situ pond measurements were taken throughout two rainy seasons of 2005 and 2006. In addition, vector density was estimated using Centers for Disease Control (CDC) miniature light traps deployed in six locations (four indoor, two outdoor) in Banizoumbou. The HYDREMATS high-resolution, coupled hydrology and entomology model of Bomblies et al. [28] was then used to successfully simulate the pool formation and daily pond water depth at the 10 m scale as well as the vector density differences between the two seasons. For a comprehensive description of the study area, the HYDREMATS simulations, and their evaluation with in situ measurements, the reader is referred to Bomblies et al. [28].

Station rainfall data for the experiments were provided by the Banizoumbou meteorological station located just outside the village. This data is available from the African Monsoon Multidisciplinary Analyses (AMMA) database (http://database.amma-international.org). To explore the potential of using satellite precipitation products to drive VECTRI on a regional scale, two satellite products, namely the Tropical Rainfall Measuring Mission (TRMM 3B42; Huffman et al. [33]) and the second version of the Famine Early Warning System (FEWS RFE2; Herman et al. [34]) were assessed as these are the two key daily rainfall products available in near real-time. The TRMM 3B42 rainfall estimates are available on a 3-hour temporal resolution and a spatial resolution of 0.25° × 0.25° between latitudes 50° north and south. Rainfall estimates from TRMM 3B42 are derived using a combination of passive microwave sensors (TMI, AMSU-B, SSM/I, and AMSR-E) and the TRMM 2A12 precipitation radar (PR) calibrated using available rain gauge data on a monthly timescale [35]. The FEWS RFE2 rainfall retrieval combines geostationary infrared information with polar orbiting microwave sensor (SSM/I and AMSU-B) data, replaced with rain gauge data where available on the global telecommunications system (GTS). This product has a spatial resolution of 0.1° × 0.1° [36]. For each product the satellite pixel was selected that contains the study area.

### VECTRI Malaria Model

#### Default model hydrology

VECTRI is an open source grid-cell distributed dynamical malaria model that operates using a resolution of the driving climate data (10–100 km). For a detailed overview the reader is referred to Tompkins and Ermert [27]. The model incorporates a simple surface hydrological parametrization scheme (V1.2.6) that estimates at each time step the fractional water coverage area in each grid cell. Fractional water coverage area is a sum of both temporary and permanent habitats. Locations with permanent year-round water bodies that can provide breeding sites can constitute transmission hot-spots, with markedly reduced seasonality in the transmission intensity, and which provide the seed to initiate transmission in neighboring areas once rains commence [37–39]. Including an estimate of the permanent breeding fraction enables the model to represent the reduced amplitude of the malaria transmission seasonal cycle in such locations.

Most of the water bodies in and around Banizoumbou are seasonal. There is one pool approximately 200 m from Banizoumbou that contains water year-round, including the dry season. However, very few larvae were found outside of the rainy season (just 5 larvae in 3 dry seasons) during two measurement campaigns during 2005 and 2006 [28]. This water body is the only watering hole in the surrounding area and is thus intensively used by villagers to water their cattle during the dry season. This results in highly polluted water during the dry season which deters *Anopheles Gambiae*. Only during the wet season, when the water quality was much improved are mosquito larvae found in abundance in the periphery of this pool. As a result of these considerations, for this study the permanent water fraction is set to zero so that only temporary ponds are accounted for.

The ground water table is usually around 30 to 50 m below the surface in the Sahel [40, 41], although there are some local exceptions, and ground water is assumed to be too deep to affect pond fraction in both models. For the study location of Banizoumbou village itself, the water table is 22 m below the surface, while for the surrounding areas the average depth is around 50 m, with a minimum depth of 10 m directly below the largest pools [42].

The net aggregated fractional water coverage of temporary pools in each grid cell was expressed as in Eq 1:

$$\begin{array}{c} \frac{dw_{pond}}{dt}=K_{w}\left(P\left(w_{max}-w_{pond}\right)-w_{pond}\left(E+I\right)\right) \end{array}$$ (1)

where *w*_*pond*_ is net aggregated fractional water coverage in a grid cell, *w*_*max*_ is maximum temporary pond coverage area, *P* is precipitation rate, *E* and *I* which were set to a fixed constant are evaporation rate and infiltration rate respectively and *K*_*w*_ is a constant that links rainfall to the growth of the temporary ponds. For details see Tompkins and Ermert [27].

The scheme is highly simplified and neglects many factors, including topographical slope, soil texture, presence of vegetation, pond geometry and heterogeneity in water infiltration rates. For example, the rate of infiltration decreases towards the middle of these temporary ponds due to the effect of clogging by sediment over time [43, 44]. This nonlinearity in infiltration relation will therefore lead to constant infiltration assumptions over- or underestimating loss of water from the ponds. The stability of ponds has also been linked with their shape. For instance, Garmendia and Pedrola-Monfort [45] observed rapid drying of cylindrical shaped ponds relative to conic shape ponds. The presence of vegetation has been documented to reduce evaporation rates of water bodies [46], although this would be challenging to represent in the scheme due to the lack of data to set the relevant parameters on a regional scale. In any case, as evaporation is generally a small loss term relative to infiltration, vegetation coverage will be of second order impact on the water balance of these water bodies.

#### Modified model hydrology

**Fig 1:**
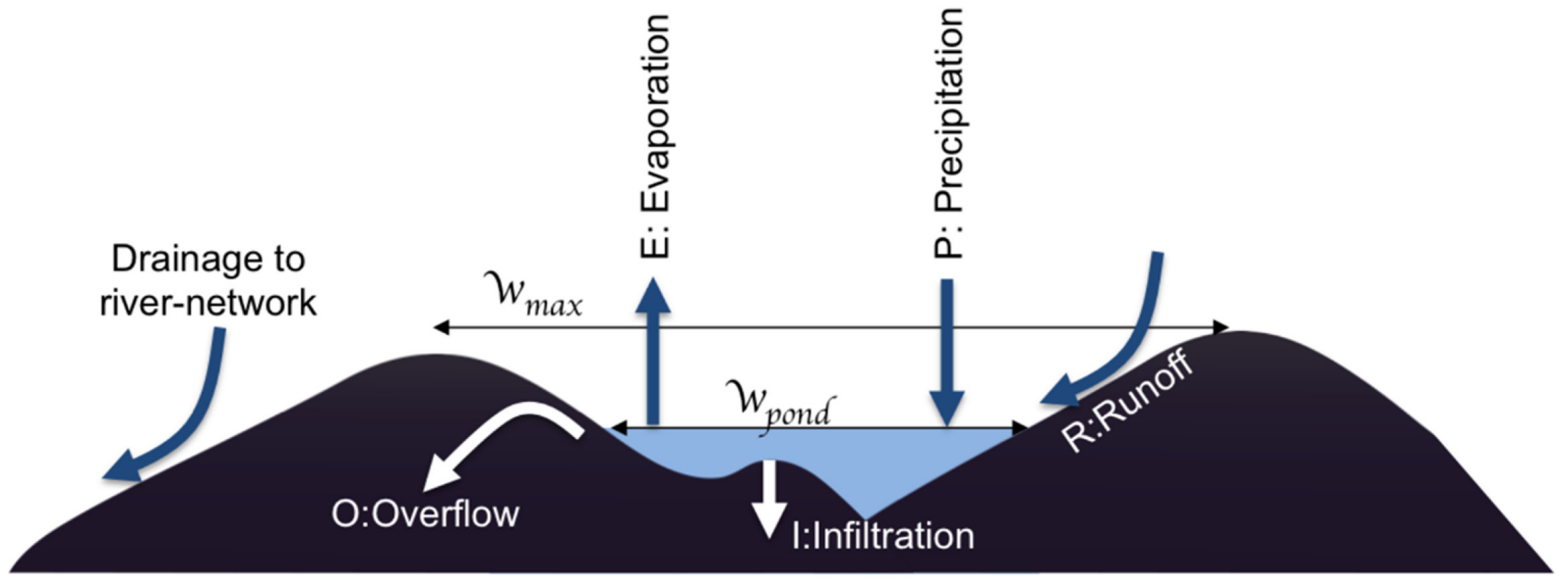
Schematic of the modified hydrology scheme adapted from Asare et al. [48]. Precipitation (*P*) is assumed to fall homogeneously across the grid-cell. In some locations the rainfall drains into stream/river catchments, while the remainder either contributes directly to pools or falls within local catchments for ponds, in which case only a proportion may reach the ponds via surface runoff. The sink of pond coverage is via the loss of water due to overflow (*O*), infiltration (*I*) and evaporation (*E*).

A new surface hydrology parametrization scheme (V1.3.0) is introduced to address some of the shortcomings of the default scheme. The modified approach is still based on the concept of small kilometer-scale catchments that cover a proportion *w*_*max*_ of a grid cell that collect water locally into temporary surface pools, while precipitation falling on the remaining area is lost to the larger scale drainage network. A schematic of the modified approach is given in Fig 1 which emphasizes that in the new model, all precipitation falling directly in ponds contributes to their growth, while in the remaining catchment area, only the surface runoff does so. Ponds lose water mass through processes of infiltration, evaporation and overflow.

In the new scheme, pool geometry is accounted for using the simple power function relation of Hayashi and Van der Kamp [47]. Unlike V1.2.6, the relationship between volume and pond fractional coverage area is also linked to the pond’s geometry in V1.3.0 as expressed in Eq 2 following Asare et al. [48]:

$$\begin{array}{c} \frac{dw_{pond}}{dt}=\frac{2}{ph_{ref}}{\left(\frac{w_{ref}}{w_{pond}}\right)}^{p/2}\left(\left(Q\left(w_{max}-w_{pond}\right)+Pw_{pond}\right)\left(1-f\right)-w_{pond}\left(E+fI_{max}\right)\right). \end{array}$$ (2)

Here *p* represents the shape factor of ponds, *h*_*ref*_ is the aggregated reference pond water depth, *w*_*ref*_ is the reference fractional coverage equated to *K*_*w*_, *Q* is the runoff (see Eq 3), *I*_*max*_ maximum infiltration which depends on the local hydrology and soil type and $f=\frac{w_{pond}}{w_{max}}$. The overflow (marked *O* in Fig 1) is modelled by a simple factor 1 − *f*. It is recalled that the pond fraction is not representative of a single water body, but rather models the statistics of a collection of water bodies. The assumption is that as the pond fraction increases, a greater number of these bodies reach their maximum capacity and lose water through overflow losses. Asare et al. [48] noted overflow losses to be significant in water bodies during the peak of the rainy season.

This hydrological scheme has been evaluated and showed good agreement with in situ pond observations [48]. Infiltration is expected to be maximum when the ponds reach their maximum surface area. For instance, in southwestern Niger, Martin-Rosales and Leduc [44] found maximum infiltration of the order of 600 mm day^−1^ after a rainfall event which reduced significantly as the water depth reached the clogging region of the pond. In the same region, Desconnets et al. [43] found a similar sharp decrease in infiltration rate from the sandy to clay-clogged area of the pond. The infiltration is modelled by a simple linear function of the pond fraction, which leads to the strong nonlinear evolution of pond fraction observed. It is emphasized that the maximum infiltration rate is only reached when the pond fraction equals the catchment area, which never occurs even after intense rainfall, and mean infiltration rates are usually an order of magnitude lower. Here, a range of suitable values for *I*_*max*_ are tested.

The *Q* term in the V1.3.0 is calculated based on the United States Department of Agriculture (USDA) USDA [49] Soil Conservation Service curve number (SCS-CN) method:

$$\begin{array}{c} Q=\frac{{\left(P-0.2S\right)}^{2}}{P+0.8S} \end{array}$$ (3a)

$$\begin{array}{c} S=\frac{25400}{CN}-254 \end{array}$$ (3b)

where *P* is rainfall (mm), *S* is potential maximum retention (mm) and *CN* (range between 0 and 100) is the curve number, a dimensionless parameter representing the land surface characteristics. When *CN* is 100, all rainfall will become runoff while all rainfall infiltrates without generating runoff when *CN* is 0. The *CN* values for various hydrological soil groups and land cover types are available from SCS-CN tables provided by the USDA [49]. Setting a suitable value for CN is a challenge. While The CN is a function of soil properties, these can be quite spatially heterogeneous and can also change in time due to surface crust formation, which can greatly increase runoff [50, 51].

The infiltration is also a function of the soil water content, which is not modelled in VECTRI to avoid increasing model complexity by the addition of further prognostic equations for multiple soil layers and the extraction of water in the root zone by vegetation. Therefore, in this study a range of appropriate values were tested assuming sandy soil with crust formation, as specified in [28, 29]. Runoff occurs when rainfall exceeds the initial abstraction capacity of the surface layer which is generally assumed to be 0.2*S*.

Hayashi and Van der Kamp [47] introduced a simple geometrical model for ponds. Studies have shown that the geometrical shape parameter in the model is approximately 2 for temporary ponds [47, 52, 53]. This simple geometrical model showed good agreement with daily observed surface area of individual micro habitats in suburb of Kumasi Ghana [48].

#### Vector model

The VECTRI model simulates the total vector density per square metre by dividing larvae into a number of development bins and vectors into a number of bins that represent the state of the gonotrophic cycle Tompkins and Ermert [27]. Temperature impacts rate of the progression of larvae through the successive growth stages, the female adult gonotrophic rates, and the mortality of both larvae and adults. The pond fraction limits larvae density through a maximum biomass carrying capacity, the value of which was adopted from the HYDREMATS model.

### HYDREMATS Malaria Model

#### Surface hydrology

HYDREMATS is a mechanistic model that simulates the pool water level and flow velocity at each time step and grid-cell based on distributed flow routing [28]. The overland flow component of the model solves the two-dimensional Saint-Venant equations (continuity and horizontal momentum equations). A finite difference solution of diffusive-wave approximations of the Saint-Venant equations is used to predict pool water depth and surface-water routing. The overland flow direction is strongly linked with variations in the slope of cell topography and surface water pools are predominantly found at topographic low points in the study region. The mean velocity of the routing is parametrized by Manning’s equation with roughness parameter depending on soil and vegetation characteristics of each cell.

The HYDREMATS land surface parametrization consists of two vegetation layers and six soil layers based on the land surface transfer scheme (LSX) of Pollard and Thompson [54]. This coupled atmosphere-vegetation-soil scheme simulates momentum, energy and water fluxes exchanges at each grid cell, and includes the partitioning of rainfall into infiltration and overland runoff.

#### Vector model

The agent-based entomological component of HYDREMATS simulates individual mosquitoes interacting with their environment as they progress through their life cycle. Flight is simulated in two dimensions using a radial random walk formulation, and entomologically important attributes are tracked for all mosquitoes (e.g. age, number of blood meals, degree-days experienced since blood meal, etc). After eclosion from one of the persistent pools, a simulated female adult mosquito begins her quest for a blood meal. She flies until she encounters a house, which is assumed to be occupied. After taking a blood meal, the mosquito rests for 24 hours, and then begins seeking suitable oviposition sites. Upon encountering a pool, the mosquito deposits a clutch of eggs in the pool and continues to repeat the cycle until she dies. In this way, characteristics of the entire mosquito population arise from the collective actions of many independent individuals. Bomblies et al. [28] gives further detail concerning the functionality of the entomology model within HYDREMATS.

### Hydrology Comparison method

**Fig 2:**
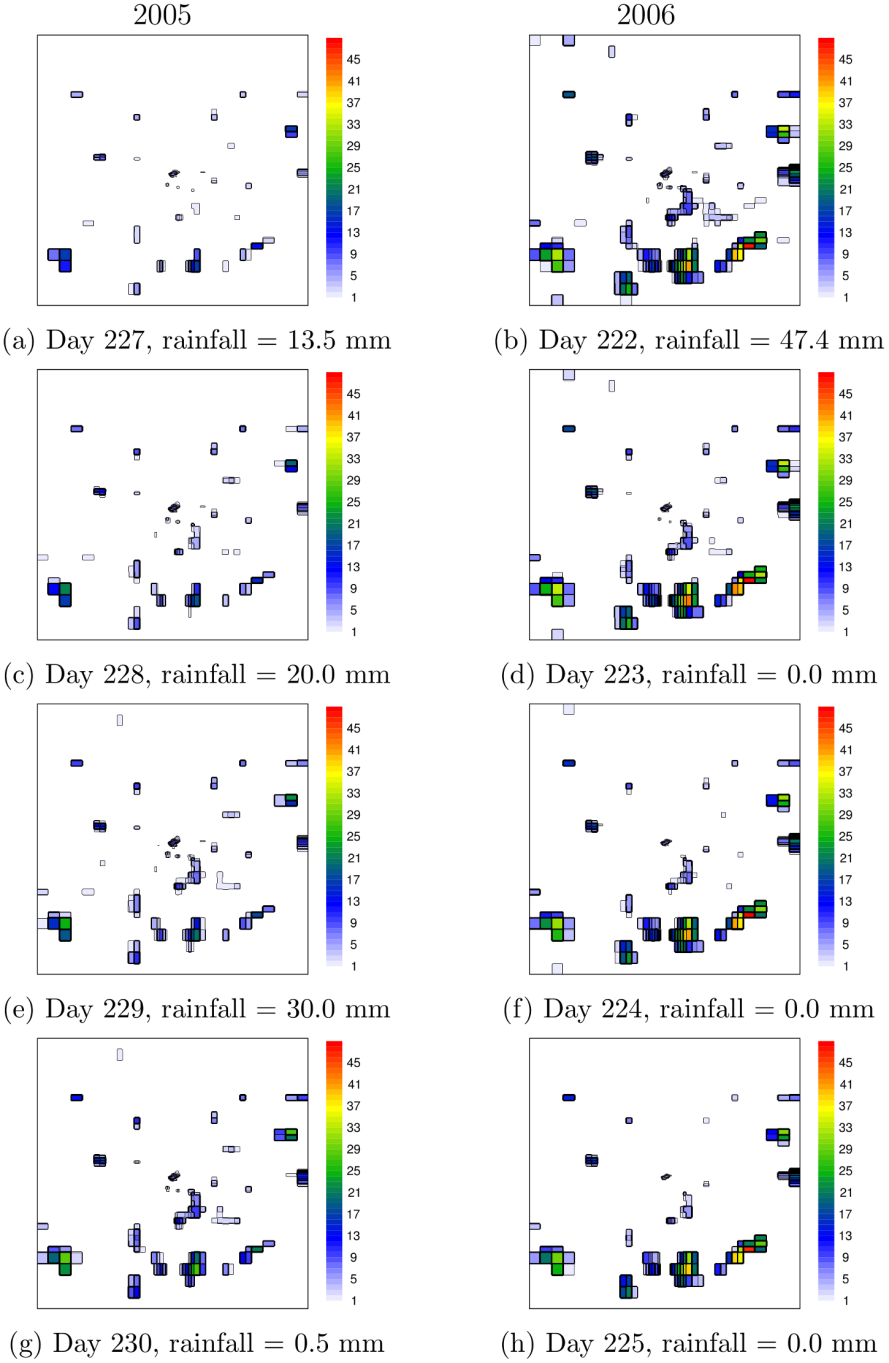
An example of daily HYDREMATS simulated water depth evolution of individual ponds over the study domain for four consecutive days in each year. The Julian day and rainfall recorded for the selected days in 2005 (left panel) and 2006 (right panel) are provided under each plot.

Assuming a 5 cm water depth threshold for each cell to be considered to contain a breeding site, HYDREMATS daily average pond coverage fraction is derived for the 2.5 × 2.5 km simulation area and used to evaluate the two VECTRI parametrization schemes which simulate a single water fraction over the study domain. Cells with water depth less than this threshold are likely to dry out within a day without rainfall and tests show little sensitivity of the results to this value (Fig 2).

In order to identify the set of parameters that minimizes the error between VECTRI and HYDREMATS hydrology schemes, a deterministic optimization based on the root mean square error (RMSE) is used. Various parameters including *w*_*max*_, *K*_*w*_, *E* + *I*, *I*_*max*_ and *CN* in the VECTRI surface hydrology schemes are perturbed from their default values. These parameters have the most influence on the pond stability on both seasonal and intraseasonal time scales. The VECTRI model is then integrated in a Monte Carlo set of ensembles using combinations of parameters. The RMSE is calculated for each experiment for the entire simulation period of 2005 to 2006.

To assess VECTRI model’s performance in simulating the water fraction and vector density, the Nash-Sutcliffe efficiency (*NSE*) [55] given by Eq 4 is used

$$\begin{array}{c} NSE=1-\frac{\sum_{i=1}^{N}{\left(S_{i}-O_{i}\right)}^{2}}{\sum_{i=1}^{N}{\left(O_{i}-O^{*}\right)}^{2}} \end{array}$$ (4)

where *S*_*i*_ is the simulated value, *O*_*i*_ is the observed value, O* is the mean of observations and N is the total number of observations. The *NSE* metric ranges between −∞ and 1 with *NSE* value of 1 indicates that observed versus simulated plot perfectly fits a 1:1 line.

## Results and Discussion

### High resolution integrations

Pond stability is determined by precipitation frequency, intensity as well as local hydrological parameters such as soil type, water table depth and micro topography. In a Sahelian environment, infiltration accounts for about 90% of the loss of water [30, 43]. Total rainfall recorded was 409.3 and 478.3 mm in 2005 and 2006, respectively falling in 44 and 39 wet days in the two years.

An example of HYDREMATS simulated water depth using station rainfall observations for the entire study region for a sequence of 4 days in 2005 and 2006 is shown in Fig 2 (see S1 Dataset). This figure illustrates pond water coverage and shows how grid cells with water depth less than 5 cm tend to be short-lived in this simulation and unlikely to survive until the next day without rainfall. Although Bomblies et al. [28] observed mosquito larvae in hoof-print of animals in this region, such habitats located far from larger scale depressions resolved by the 10 m model resolution tended to be short-lived, as persistence times were only several hours and therefore far less than the approximately seven days required under optimum conditions for completion of the mosquito aquatic stage development [24]. In contrast, animal hoof-prints located within the catchment of large depressions were stable enough to produce adult mosquitoes. Thus productive developmental habitats cluster around topographical low areas [28]. The locations of the HYDREMATS simulated habitats are similar for the two years but with differences in habitat extent and stability (Fig 2).

### VECTRI hydrology parametrization Evaluation

**Fig 3:**
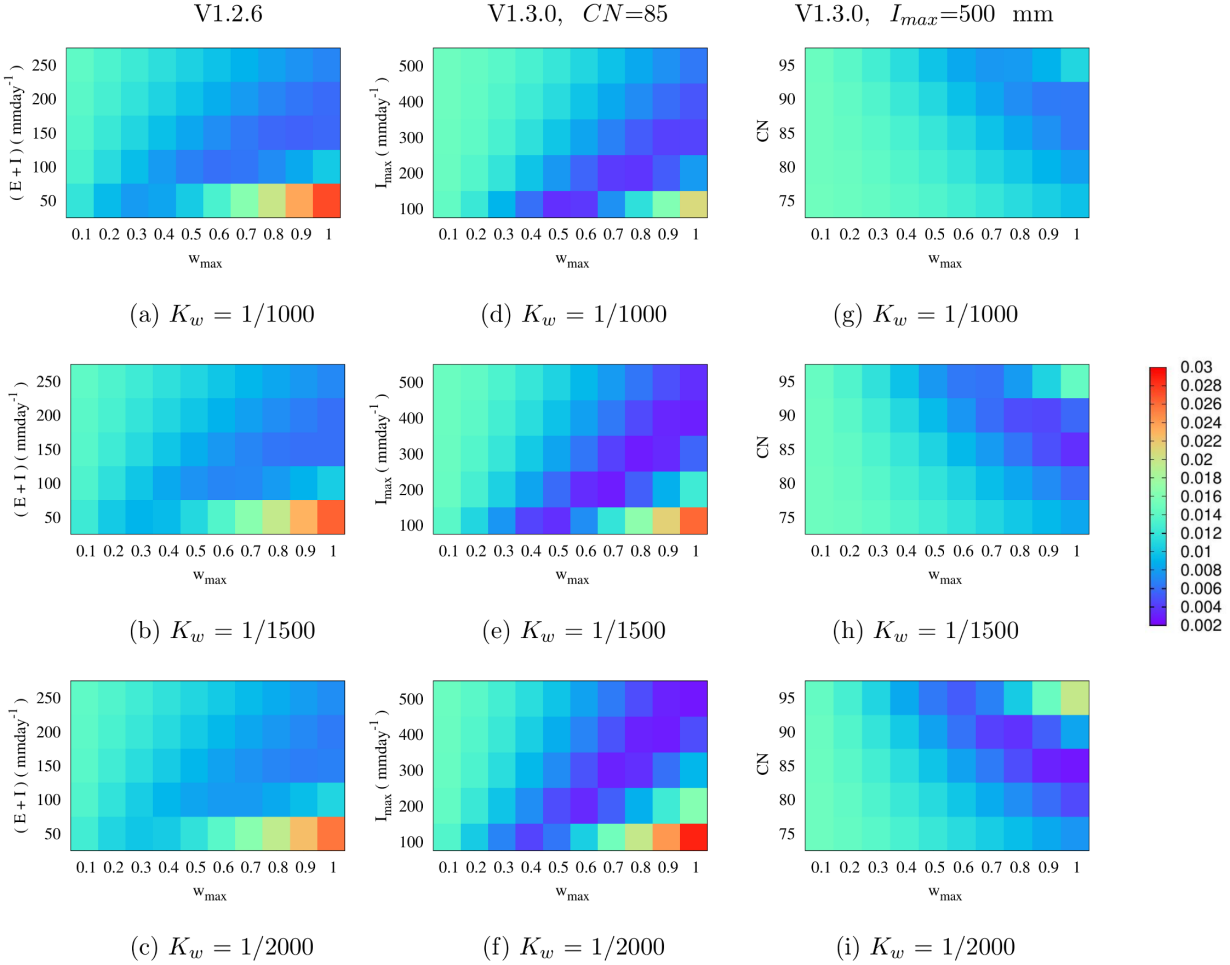
RMSE between HYDREMATS and VECTRI schemes (with varying tunable parameters) simulated daily water fraction. Left panel: VECTRI hydrology V1.2.6, middle panel: V1.3.0 varying maximum infiltration *I*_*max*_ with a constant *CN* of 85 and right panel: V1.3.0 varying *CN* with constant *I*_*max*_ of 500 mm day^−1^.

Fig 3 (see S2 Dataset) shows the pond fraction root mean square error (RMSE) for VECTRI (V1.2.6 and V1.3.0) using a combination of various tunable parameters as described in the methods section. Regarding V1.2.6 (Fig 3*a*, 3*d* and 3*g*), decreasing *K*_*w*_ resulted in an increase in RMSE with respect to HYDREMATS with the exception of when *E* + *I* = 50 mm day^−1^. Although decreasing *K*_*w*_ caused a decrease in pond growth for a given rain rate, at the same time it increases pond stability by reducing daily loss of water from the pond due to the linear relation between *K*_*w*_ and rainfall and *E* + *I* terms. Increasing the constant loss term *E* + *I* reduces lifespan of the ponds as expected. Taking an *E* + *I* value of 100 or 150 mm day^−1^ reduces the RMSE between V1.2.6 and HYDREMATS with *w*_*max*_ of 0.5. In this case, during intense rainfall events when the pond water extends to the sandy porous fringes associated with high infiltration, the constant infiltration rate will lead to an underestimation of the total infiltrated water.

Smaller RMSE values were observed between HYDREMATS and VECTRI V1.3.0 with constant *CN* = 85 (Fig 3*b*, 3*e* and 3*h*) at smaller *w*_*max*_ compared to V1.2.6. Although varying *I*_*max*_ affects the values of *w*_*max*_ that resulted in good agreement with HYDREMATS, the range of values for *w*_*max*_ that produce lower RMSE are smaller relative to the V1.2.6. The primary reason is the scaling factor *f* that moderates infiltration by accounting for increases or decreases in daily infiltration rates with pond water extent [43, 44]. In addition, both the runoff and *K*_*w*_ coefficient in V1.3.0 are also nonlinear. The V1.3.0 scheme with fixed maximum infiltration (*I*_*max*_ = 500 mm day^−1^) also shows a similar pattern to the previous V1.3.0 experiment but requires large values of *w*_*max*_ to minimize the RMSE (Fig 3*c*, 3*f* and 3*i*). The good agreement between V1.3.0 (*I*_*max*_ = 500 mm day^−1^) and HYDREMATS at high values of *w*_*max*_ demonstrates that the set infiltration threshold is likely to be too high for the study region.

It is clear from these experiments that there is no unique set of parameters which leads to a good performance of the VECTRI schemes, and many parameter combinations enable the VECTRI surface hydrology schemes to reproduce water fraction similar to that of the HYDREMATS model. This is due to the use of several calibration parameters to improve the performance of a single model metric; the higher degrees of freedom in the tuning parameter set leading to model equifinality. Beven discusses this in the context of hydrological models [56], but it is common to any (especially nonlinear) modelling system with multiple uncertain parameters. For example, many thousands of a climate model parameter settings produced present-day simulations of global mean temperature deemed an acceptable representation of reality in the climateprediction.net project [57, 58].

The VECTRI new parametrization V1.3.0, especially with fixed *CN*, showed good agreement with HYDREMATS at lower *w*_*max*_ values relative to V1.2.6. In addition, although the VECTRI surface hydrology schemes are considerably less complex relative to HYDREMATS, the model parameters closely mimic processes they are meant to represent as seen in Fig 3.

**Fig 4:**
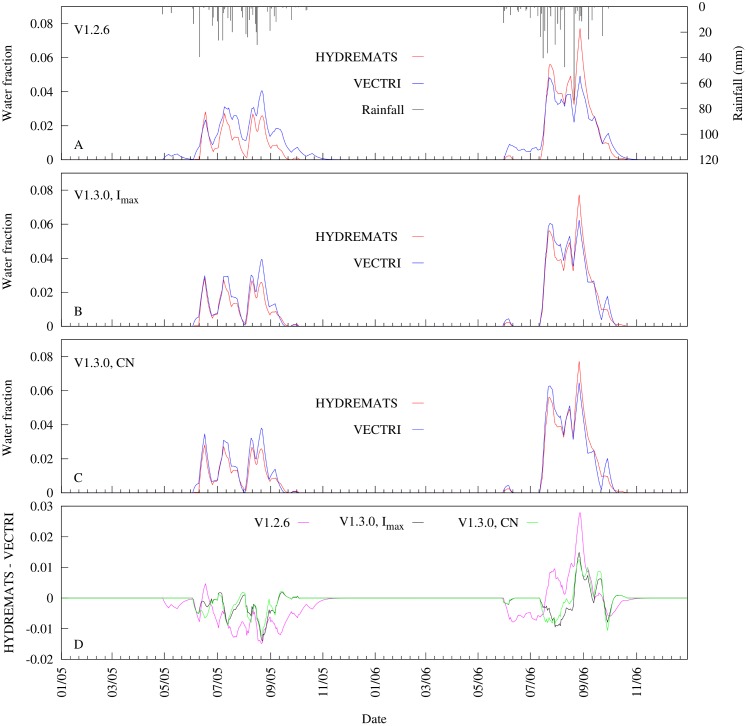
Comparison of 7-day moving average time series of simulated water fraction from HYDREMATS and VECTRI schemes. The VECTRI schemes time series were generated from a sets of VECTRI calibrated parameters that minimize RMSE values with respect to HYDREMATS. (a) V1.2.6, (b) V1.3.0 varying maximum infiltration (*I*_*max*_) assuming a constant *CN* of 85, (c) V1.3.0 varying *CN* at a constant *I*_*max*_ of 500 mm day^−1^, and (d) the difference between HYDREMATS and VECTRI.

Fig 4 shows an example of a 7-day moving average time series of simulated water fraction by the HYDREMATS and the two VECTRI schemes using combinations of parameters that resulted in the lowest RMSE with respect to HYDREMATS (Table 1; see S3 Dataset). The 7-day window was selected because it is the optimum time for successful completion of aquatic stage mosquito development [24]. The simulation results clearly demonstrate that the variability in the daily water fraction follows trends in rainfall relating to its inter-storm period, intensity, and frequency. There was a slight increase in recorded rainfall of about 69 mm (16%) from 2005 to 2006 with 2006 having shorter storm return period compared to 2005. The impact of rainfall variability on the model’s simulated daily pond fraction over the two year period highlights that rainfall sub-seasonal variability can be as important for transmission intensity as seasonal totals within the Banizoumbou village. Whereas greater inter-storm periods may have little influence on the stability of permanent and semi-permanent ponds, they will cause desiccation of temporary ponds [30].

**Table 1:** The VECTRI schemes set of tuning parameters that lead to the minimum RMSE between HYDREMATS and VECTRI simulated water fractions.

VECTRI scheme	Set of tunable parameters
V1.2.6	*K*_*w*_ = 1/1000, *w*_*max*_ = 0.9, *E* + *I* = 150 *mmday*^−1^
V1.3.0 (*CN* = 85)	*K*_*w*_ = 1/1000, *w*_*max*_ = 0.7, *I*_*max*_ = 200 *mmday*^−1^
V1.3.0 (*I*_*max*_ = 500 *mmday*^−1^)	*K*_*w*_ = 1/1500, *w*_*max*_ = 1, *CN* = 85

Fig 4 further reveals that despite the simplicity of the VECTRI surface hydrology parametrization schemes, it is able to reproduce the fractional water coverage evolution as simulated by the HYDREMATS model, although the V1.3.0 scheme shows a further improvement (*NSE* = 0.95) relative to the V1.2.6 scheme (*NSE* = 0.85). The initial ponding occurring in the V1.2.6 scheme in 2005 as a result of two isolated rainfall events of order of 6 mm is absent in both the V1.3.0 and the HYDREMATS. Clearly in the area like Banizoumbou, these events are unlikely to cause ponding especially occurring at the onset of the monsoon season. However, the VECTRI model tends to overestimate and underestimate the HYDREMATS water fraction in 2005 and 2006, respectively. A possible reason for this disparity may be the different response of the two VECTRI schemes to different rainfall pattern. The relative magnitude of the water fraction predicted by the two models changes as the season progresses. This is particularly apparent with VECTRI V1.2.6 in the 2006 season, where VECTRI predicts larger water fraction during the season onset, and lower fractions later in the season. Part of the disparity is due to the simple approach of the earlier surface hydrology scheme that does not account for run off. The rainfall events during the earlier season of 2006 are small in magnitude, and by introducing the curve-number based runoff scheme V1.3.0 prevents these events from producing breeding sites, in agreement with HYDREMATS. Nevertheless, further improvements could be made by including soil moisture, which would increase infiltration in the earlier season when soil moisture is dry and reduce it in the later season, albeit at the cost of increasing the complexity of the scheme.

Furthermore, the difference in observed rainfall between the two years (see rainfall in Fig 4) caused the simulated pond water fraction by both models to have a higher mean water fractional coverage in 2006 relative to 2005, more than expected by 117, 15, 72 and 75% for HYDREMATS, V1.2.6, V1.3.0 (fixed *CN*) and V1.3.0 (fixed *I*_*max*_) respectively.

**Fig 5:**
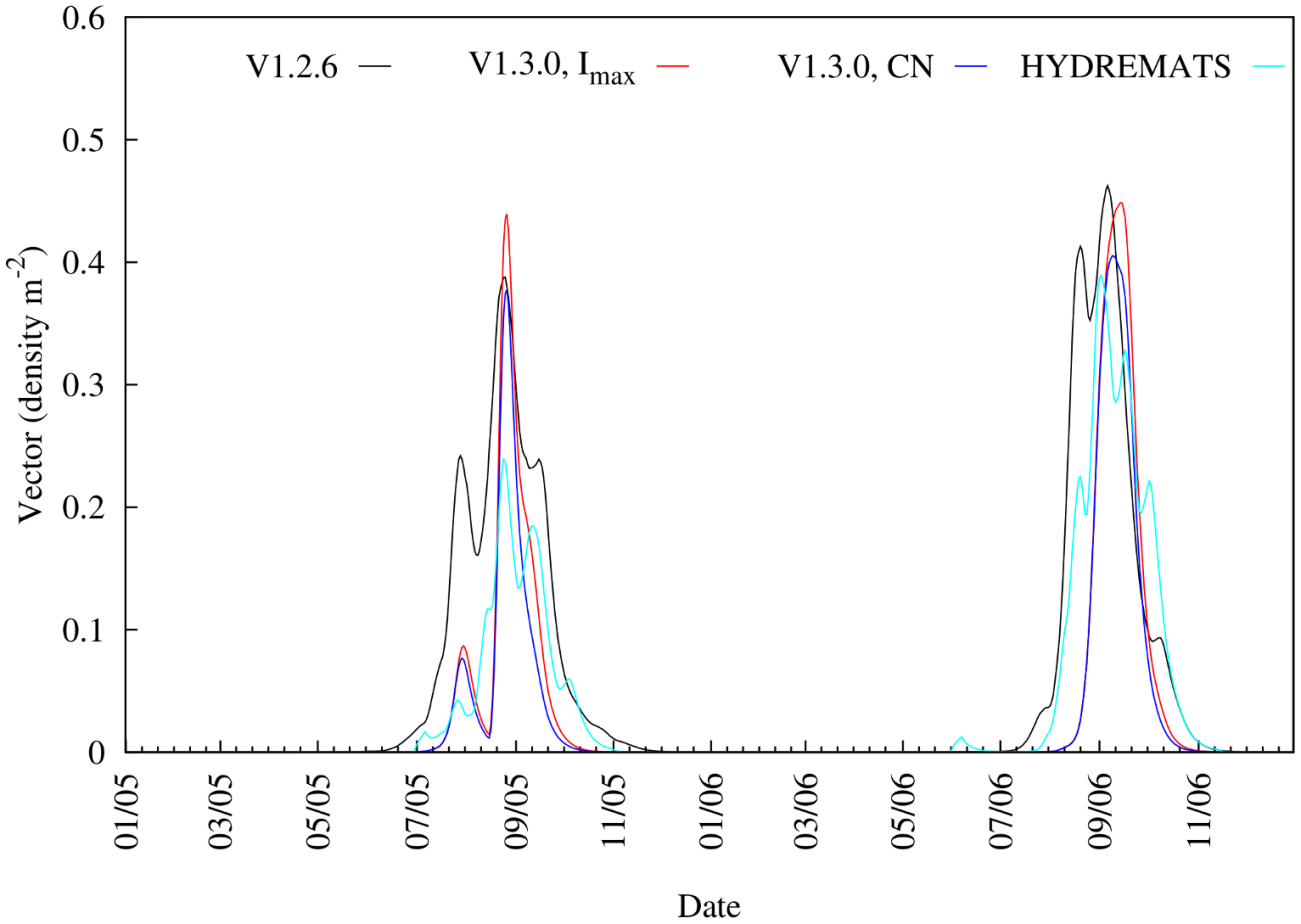
Comparison of 7-day moving average time series of simulated mosquito vector abundance from HYDREMATS and VECTRI schemes. For the VECTRI experiments, identical calibrated parameters as for Fig 4 were used.

This variability in daily simulated water fraction due to the influence of rainfall also impacts vector abundance. For example, in this same study area, Bomblies et al. [28] captured 140% more mosquitoes at the same locations in the village in 2006 than in 2005. The results from the simulated mosquito vector abundance by both models are consistent with the observed increase in 2006 relative to 2005 (Fig 5; S4 Dataset) but with a wide range of increases. For instance, HYDREMATS simulates a 84% increase between 2005 and 2006, while the three versions of VECTRI, V1.2.6, V1.3.0 (fixed *CN*) and V1.3.0 (fixed *I*_*max*_), produced increases of 4, 54 and 36%, respectively. The differences in the VECTRI simulated vectors densities are due only to the changes in the surface hydrology scheme as all other model components are identical in the simulations, highlighting the critical importance of the hydrological component of the model, that is possibly the least constrained by observations and thus likely to be one of the key contributors to malaria model uncertainty. In particular, it is seen that the V1.2.6 suffers from a too early onset, particularly in 2005, due to its neglect of the runoff process, which resulted in light rains causing pooling in the pre-onset phase of the monsoon (Fig 5). From the two VECTRI schemes, larvae density simulated from the revised scheme showed better agreement (*NSE* = 0.70) with the HYDREMATS relative to the default scheme (*NSE* = 0.54). In fact, considering the simplicity of the bulk larvae/vector schemes in VECTRI relative to the highly detailed agent based approach employed in HYDREMATS, and the fact that no parameter tuning of these components was performed (although it is recalled that the biomass carrying capacity parameter in VECTRI is adopted from HYDREMATS), the similarity between the conversion of water fraction to vector density by the two models is quite remarkable. This result supports the conjecture that with knowledge of the factors that affect surface hydrology, a simple model such as VECTRI can potentially be employed to simulate vector density and subsequently malaria transmission on a local scale.

### VECTRI simulation with satellite products

**Fig 6:**
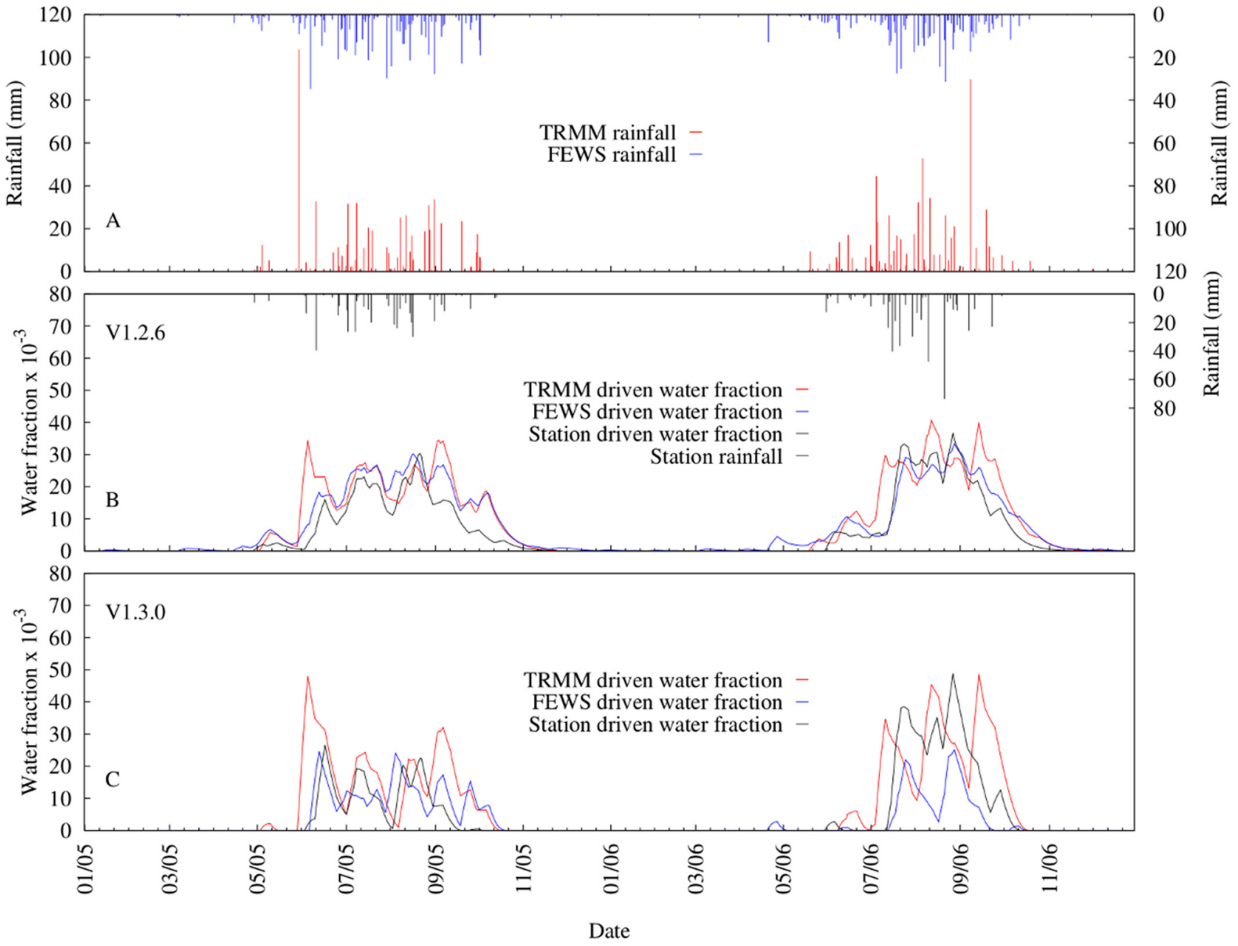
Comparison of 7-day moving average time series between VECTRI simulated water fraction driven by station rainfall, TRMM 3B42 and FEWS RFE2 rainfall estimates. (a) TRMM and FEWS RFE2, (b) V.1.2.6 scheme predicted water fraction and (d) V.1.3.0 scheme predicted water fraction.

Over the two year study period, TRMM 3B42 and FEWS RFE2 recorded 1305.5 and 1181.4 mm of rainfall, about 47 and 33% respectively more than the station measurement records. Fig 6 shows the 7-day moving average time series of VECTRI (both V1.2.6 and V1.3.0 schemes) driven by both rainfall estimates and station observation simulated water fraction. The differences in the rainfall also impact the simulated water fractions. For example, the mean simulated water fraction over the study period increased about 45 and 49% for V1.2.6 and V1.3.0 driven by TRMM 3B42 rainfall respectively, when compared to station runs. Interestingly, the FEWS RFE2 mean simulated water fraction was about 34% more than station runs for V1.2.6 but about 25% less than that of station runs for V1.3.0. One reason for this disparity is that in V1.2.6 rainfall is used directly as input to drive the model while rainfall is converted to runoff in V1.3.0. To a certain extent, the initial abstraction term in runoff computation [49] sets the threshold below which rainfall amount generates no runoff. For instance, when setting *CN* = 85 in V1.3.0, rainfall less than ≈ 9 mm will produce no runoff and leads to no increase in the water fraction but some increase in the water fraction occurs in V1.2.6. In addition to this, the runoff term in V1.3.0 is highly nonlinear and so greater recharge occurs with intense rainfall events. Lastly, there is also the fact that infiltration in V1.3.0 adds another nonlinearity and therefore contributes to the different simulated water fraction results from the two schemes.

The relatively good agreement between simulated water fractions using station data versus the two satellite retrievals, although VECTRI driven by FEWS RFE2 (V1.2.6: *NSE* = 0.83; V1.3.0: *NSE* = 0.64) performed reasonably well compared to TRMM ((V1.2.6: *NSE* = 0.55; V1.3.0: *NSE* = 0.47). This further reveals that there were few heavy rainfall events recorded by the station that were missed by FEWS RFE2 or TRMM 3B42. It is the intense rainfall events that contribute most to ponding and thus greatly influences the simulated water fraction as shown in Fig 6. Light rainfall events that the satellites may miss are less important for ponding. The results clearly show that the greatest disparities between VECTRI simulated water fractions occur on days when either TRMM 3B42 or FEWS RFE2 record high rainfall amount but little or no rainfall is recorded by the ground station or vice versa.

Another important finding of the VECTRI model driven by TRMM 3B42 is the ability of the surface hydrology scheme to simulate sub-seasonal rainfall variability impacts on both water fraction dynamics and vector abundance. TRMM 3B42 recorded almost the same amount of rainfall in 2005 (653.1 mm) and 2006 (652.4 mm) but with a variable sub-seasonal pattern (see TRMM 3B42 rainfall in Fig 6). The VECTRI simulated water fraction and mosquito vector abundance showed a difference of about 18 and 43% respectively, for V1.3.0 and 1 and 5% respectively, for V1.2.6.

### Conclusions

The challenge of validating surface hydrology parametrizations of dynamical malaria models arises from the lack of data from both field observations or remote sensing techniques due to the small spatial scales of the key malaria vector habitats. To partially address this, the HYDREMATS high resolution village-scale model that explicitly simulates individual ponds was used to provide a proxy for high resolution observations of breeding sites and used to evaluate the performance of the bulk parametrization scheme for water fraction used in the regional-scale malaria model, VECTRI. In addition to the default scheme, a modified scheme is proposed that accounts for pond geometry more realistically and also incorporates the nonlinearities of the surface runoff and the infiltration processes.

The results reveal that both VECTRI surface hydrology schemes were able to reproduce seasonal and intraseasonal variability in pond water fraction (*NSE* > 0.85), with the modified scheme able to produce a closer match to the explicit benchmark model, HYDREMATS. However, the default VECTRI model tended to overestimate and underestimate the HYDREMATS water fraction in 2005 and 2006, respectively, and overestimate water fraction early in the rainy season. Accounting for run-off processes in a revised scheme improved this bias, and lead to more accurate predictions of the ponding onset at the start of the rainy season, although it is possible that further improvement could be made by representing soil moisture in the model, albeit at the cost of greatly increasing the model complexity and numerical cost. The results indicate that, after calibration, a simple scheme is able to represent the evolution of the ensemble statistics of small-scale breeding sites.

Numerous malaria endemic regions are characterized by inadequate ground observations of rainfall and thus the impact of replacing the local ground-based station measurements with remotely sensed retrievals of rainfall from FEWS RFE2 and TRMM 3B42 was assessed. Despite the contrasting scales of the measurements and the uncertainties related to the retrieval algorithms, the study showed that satellite data could nevertheless produce a reasonable simulation of the sub-seasonal evolution of the pond fraction for this area.

While the modified VECTRI hydrology parametrization presented here represented an improvement of the default scheme, further steps are required to enhance the performance of the model when applied on a regional scale. In addition to the representation of soil moisture, outside the Sahel, in locations where the water table approaches the surface towards the end of the rainy season, the model will likely underestimate water fraction due to the neglect of this process. Moreover, the present neglect of topography means that the model is unable to account for differences between flat or steeply sloping terrain. Valley bottoms, in particular with flat terrain, have been identified as focal points for malaria transmission, for example [59]. The present work shows the strong potential of the simple modelling approach if the model is calibrated, but the model using the default parameters was subject to considerable errors in the mean pond coverage, even if the sub-seasonal variability was broadly similar. Another goal is thus to develop a spatial calibration methodology. This may be addressed using ultra-high resolution hydrological retrievals from the latest generation of satellites such as the recently launched Sentinel 1, or possibly by attempting to calibrate the malaria model output parameters using information such as district case data, however, it is clear that this calibration step represents a considerable challenge.

## Supporting Information

S1 Dataset A complete HYDREMATS simulated daily water depth for the entire study area and period. These text files are named according to each day using Julian day numbers.
(GZ) Click here for additional data file.

S2 Dataset A complete set of VECTRI simulated outputs (both default and modified schemes) for various range of tuning parameters. These NetCDF files can be viewed using ncview.
(GZ) Click here for additional data file.

S3 Dataset Files containing HYDREMATS and VECTRI (using set of parameters given by Table 1) simulated water fractions. (GZ)

S4 Dataset Files containing HYDREMATS and VECTRI (using set of parameters given by Table 1) simulated vector density. (GZ)
